# Extremely Preterm Infants Have a Higher Fat Mass Percentage in Comparison to Very Preterm Infants at Term-Equivalent Age

**DOI:** 10.3389/fped.2020.00061

**Published:** 2020-03-10

**Authors:** Marlies Bruckner, Zahra Khan, Christoph Binder, Nicholas Morris, Bernadette Windisch, Sandra Holasek, Berndt Urlesberger

**Affiliations:** ^1^Division of Neonatology, Department of Pediatrics and Adolescent Medicine, Medical University of Graz, Graz, Austria; ^2^Department of Food Science and Human Nutrition, University of Veterinary and Animal Sciences, Lahore, Pakistan; ^3^Division of Neonatology, Pediatric Intensive Care Medicine and Neuropediatrics, Department of Pediatrics and Adolescent Medicine, Medical University of Vienna, Vienna, Austria; ^4^Division of Immunology and Pathophysiology, Otto Loewi Research Centre, Medical University Graz, Graz, Austria

**Keywords:** nutrition, body composition, air displacement plethysmography, PEA POD, preterm, fat mass, weight percentile, gestational age

## Abstract

**Background:** Early nutritional support of preterm infants is important because it influences long-term health and development. Body composition has an influence on cardiovascular disease, metabolic syndrome, and neurocognitive outcome in the long term.

**Objective:** To assess body composition in preterm infants <32 weeks of gestation at term-equivalent age and to analyze the influence of an optimized nutritional approach.

**Methods:** This is a prespecified secondary outcome analysis of a prospective observational study comparing the body composition in regard to gestational age. The preterm infants were classified according to gestational age as extremely preterm infants (<28 weeks gestation at birth) and very preterm infants (≥28 weeks gestation at birth) and according to weight percentile as appropriate for gestational age and small for gestational age. Body composition was determined by air displacement plethysmography using the PEA POD. The preterm infants obtained nutrition according to the ESPGHAN 2010 Guidelines.

**Results:** Seventy-four preterm infants were analyzed. The mean (SD) gestational age was 28.7 (2.4) weeks, and birth weight was 1,162 (372) g. Fat mass percentage was significantly higher in extremely preterm infants in comparison to very preterm infants [17.0, 95% confidence interval (CI) 15.9–18.1 vs. 15.5, 95% CI 14.7–16.2]. There was no significant difference of fat mass percentage according to weight percentiles.

**Conclusions:** Extremely preterm infants had a significantly higher fat mass percentage compared to very preterm infants at term-equivalent age. There was no significant difference of fat mass percentage according to weight percentiles.

## Introduction

Adequate early nutritional support of preterm infants is essential for long-term health and development (1). Especially, postnatal growth restriction of extremely preterm infants still remains a big challenge worldwide and is associated with an impaired neurodevelopmental outcome (2, 3). Evaluation of the body composition and especially fat mass (FM) gain seems to be more accurate to evaluate the nutritional status in comparison to anthropometric parameters including Z-scores (4, 5). The optimal body composition and reference values in ex premature infants at term-equivalent age are still unknown (6, 7). Extreme prematurity is a risk factor for the development of adiposity (8) and, especially, small for gestational age (SGA) infants, who have undergone rapid catch up growth, have been reported to show an altered insulin resistance, and body composition with an increase in fat mass percentage (FM%) (8–10). On the other hand, a recent longitudinal observational study showed that a higher fat mass in preterm infants under 32 weeks of gestational age was related to an improved neurodevelopment outcome at the age of 18 months (11). Furthermore, in previous studies, premature infants with a weight Z-score under −2 SD at term equivalent age showed reduced FM% values, raising some concern that neurocognitive outcome may be impaired in these infants in later life (12). However, studies evaluating body composition and especially FM in extremely premature infants at term equivalent age are limited.

The aim of the study was to assess FM and FFM in very preterm infants (VPI) born ≥28 weeks in comparison to extremely preterm infants (EPI) born <28 weeks of gestation at term-equivalent age. The second aim of the study was to analyze the relationship between FM and weight percentiles in neonates, who received nutrition according to the ESPGHAN 2010 Guidelines (13, 14).

## Methods

This study is a prespecified secondary outcome analysis of a prospective observational study conducted at the Division of Neonatology, Medical University of Graz, Austria (15). Preterm infants born <32 weeks gestation who were admitted to the neonatal intensive care unit between March 2014 and May 2015 were included. Exclusion criteria were the presence of congenital malformations, abdominal surgery, genetic syndromes, and metabolic disorders.

The present analysis and PEA POD measurements were a standard procedure during the period of that nutritional analysis study (15). The study was approved by the ethics committee of the Medical University of Graz, with written parental consent prior to inclusion.

### Body Composition

Body composition was performed by air displacement plethysmography, using the PEA POD, Infant Body Composition System (Cosmed Inc., Concord, MA, USA), which is an accurate and reliable technique considered as the international gold standard for non-invasive evaluation of body composition in infants (16, 17). The PEA POD measurements were performed as close as possible to term-equivalent age before discharge at a time when the infants no longer needed any form of respiratory or circulatory support, had not any kind of vascular access, and were stable enough to cease monitoring during the measurements. The PEA POD measurements were part of a standard procedure during the period of the above nutritional analysis study by Khan et al. (15).

To measure whole body volume, the device is equipped with two chambers of identical volume, the test chamber, and the reference chamber separated by a volume-perturbing diaphragm sensitive to pressure changes. First, the PEA POD calculates the body surface area using the Boyd formula, which is then used to obtain the surface area artifact. The surface area artifact and the thoracic gas volume, which is a predicted value, are needed to correct the raw body volume, measured by the PEA POD. With these variables, body volume can be calculated. Electronic scales are included in the PEA POD to measure the infants mass. By using body mass and volume, the whole body density can be calculated. Fat mass can easily be calculated by knowing the density of fat, which is accepted as a constant of 0.9007 g/ml. The density of FFM changes during growth and depends on age and sex. The PEA POD uses age- and sex-specific density of FFM values, which are available in literature (18, 19). Fat-free mass is the calculus of the total body mass minus the fat mass (20–22). The PEA POD can be used from a weight of 1 kg up to 8 kg and uses a two-compartment model to calculate FM and FFM (18, 19).

### Length and Weight

At birth, infants were weighed using electronic scales (Soehnle Scales CWB 7726, made in Germany). At body composition measurement, infants were weighed using the integrated scales of the PEA POD device. All measurements of length were performed using the SECA 210 Mobile Measuring Mat for babies and toddlers (Vogel & Halk GmbH & Co Hamburg, Germany) (15).

### Feeding Regimen

The local feeding protocol of the included preterm infants has previously been described in detail by Khan et al. (15). The preterm infants in the present study obtained nutrition according to the ESPGHAN 2010 Guidelines (13, 14). In the first week after birth, the average macronutrients and energy supply were 2.44 g/kg/day of proteins, 3.39 g/kg/day of lipids, and 72 kcal/kg/day (15). In the second week after birth, the preterm infants already received 3.26 g/kg/day of proteins, 5.60 g/kg/day of lipids, and 120 kcal/kg/day (15).

### Statistics

For the first analysis, the preterm infants were stratified according to their gestational age: extremely preterm infants (EPI group) born <28 weeks gestation and very preterm infants (VPI group) born ≥28 weeks gestation. For the second analysis, preterm infants were stratified according to their weight percentile (SGA = small for gestational age or AGA = appropriate for gestational age) at the time point of birth and measurement, making up four groups (AGA/AGA, AGA/SGA, SGA/SGA, SGA/AGA). The classification was based on a publication of the ESPGHAN and on a consensus paper of the Austrian Society for Pediatric and Adolescent Medicine (ÖGKJ) (1, 23). To determine which neonate was small for gestational age, we used the 10th centile of the “Fenton 2013 Growth Calculator for Preterm Infants.” Weight percentiles and z-scores were calculated using the Fenton 2013 online percentile calculator (http://ucalgary.ca/fenton). The data from the individual measurements were collected in a Microsoft Excel spreadsheet and edited. Mann–Whitney U-Test and Kruskal–Wallis test was used in intergroup comparisons of categorical variables, and categorical variables were expressed as numbers and percentages. Multivariable linear regression was used to examine the association between the FM and FFM and the study groups with adjustment for the covariates: sex (24) and gestational age at measurement (25). Estimated adjusted means and adjusted mean differences with 95% confidence intervals (CI) are reported. A value of *p* < 0.05 was considered statistically significant. The calculations were performed using the IBM SPSS Statistics 23 software.

## Results

### Study Population

**Figure 1:**
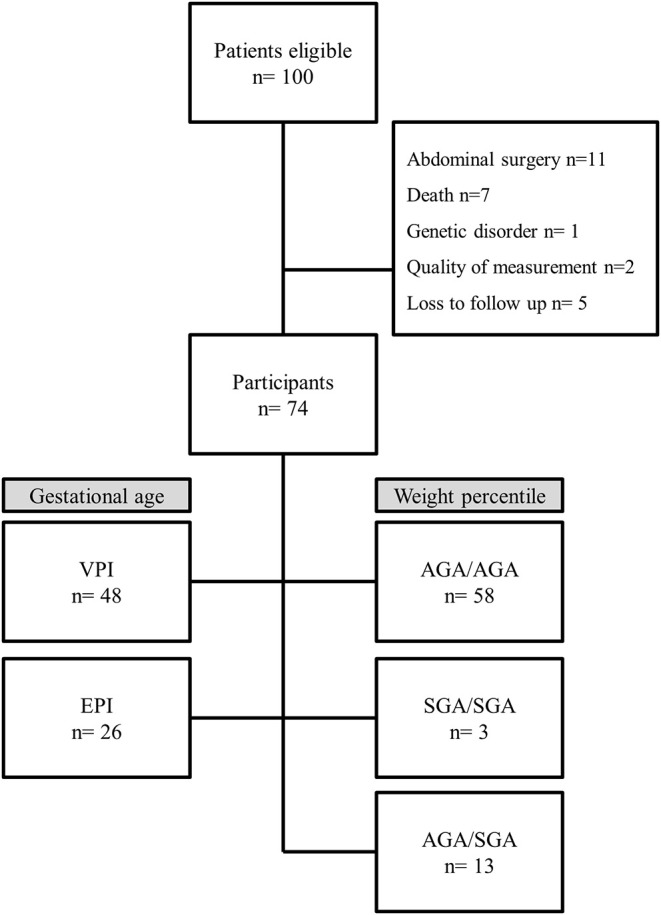
Flow chart. VPI, very preterm infants ≥28 weeks gestation; EPI, extremely preterm infants <28 weeks gestation; AGA/AGA, born and discharged appropriate for gestational age; SGA/SGA, born and discharged small for gestational age; AGA/SGA, born appropriate for gestational age and discharged small for gestational age.

One hundred preterm infants born <32 weeks of gestation were admitted to the neonatal intensive care unit during the study period. Twenty-six were excluded for the following reasons: abdominal surgery (*n* = 11), death before body composition measurement (*n* = 7), genetic disorder (*n* = 1), insufficient quality of measurement (*n* = 2), and loss to follow-up (*n* = 5) (Figure 1). A total of 74 preterm infants were included. In regard to gestational age, the infants were assigned to the following groups: VPI group: 48 infants (65%) and EPI group: 26 infants (35%) (Figure 1).

In regard to growth percentiles, the infants were assigned to the following groups: AGA/AGA: 58 infants (78%); group SGA/SGA: 3 infants (4%), and group AGA/SGA: 13 infants (18%). No infant could be assigned to group SGA/AGA (Figure 1). Preterm infant's characteristics are described in detail in Table 1. Mean FM% of the whole study cohort was 15.9 (SD 3.7).

**Table 1:** Demographic data and outcome parameter for all infants and according to the different stratifications.

	**All subjects (*n* = 74)**	**VPI (*n* = 48)**	**EPI (*n* = 26)**	***p*-value**	**AGA/AGA (*n* = 58)**	**AGA/SGA (*n* = 13)**	**SGA/SGA (*n* = 3)**	***p*-value**
**Data at birth**								
Birth weight (g)	1,162 ± 372	1,370 ± 279*	779 ± 149*	<0.001	1,254 ± 342	928 ± 314*	705 ± 284*	0.019^c^
z-score weight	−0.4	−0.4	−0.5	0.552	−0.1*	−0.9*	−1.5*	<0.01^b^, ^c^
Gestational age (week)	28.7 ± 2.4	30.2 ± 1.2*	26.1 ± 1.3*	<0.001	29 ± 2.2	27.9 ± 3.0	27 ± 3.2	0.300
Male (*n*; %)	37; 50	22; 46	15; 42	0.333	28; 48	6; 46	3; 100	0.212
**Data at discharge**								
Postnatal age at discharge (week)	36.8 ± 2.6	35.8 ± 1.6*	38.6 ± 3.2*	<0.001	36.2 ± 2.1*	38.6 ± 2.9*	41.5 ± 4.5*	0.001^a^, ^b^
Length of stay (day)	56 ± 31	39 ± 15*	89 ± 28*	<0.001	50 ± 25*	74 ± 39*	101 ± 45*	0.017^a^, ^b^
Weight at discharge (g)	2,438 ± 397	2,290 ± 281*	2,712 ± 446*	<0.001	2,414 ± 368	2,432 ± 410	2,917 ± 710	0.384
z-score weight	−1.0	−0.9	−1.1	0.172	−0.8*	−1.7*	−1.6*	<0.03^b^, ^c^
Length at discharge (cm)	44.9 ± 2.4	44 ± 2.4*	46 ± 2.2*	0.009	45.1 ± 2.6	44 ± 2.2	44.9 ± 3.2	0.464
Age at measurement (day)	56 ± 31	39 ± 15*	89 ± 28*	<0.001	50 ± 25	74 ± 39	101 ± 45	0.212

Data presented as mean ± SD and n (%). *Significant difference:

a Significant difference between AGA/AGA and AGA/SGA;

b Significant difference between AGA/AGA and SGA/SGA;

c *Significant difference between AGA/SGA and SGA/SGA. VPI, very preterm infants ≥28 weeks gestation; EPI, extremely preterm infants <28 weeks gestation; AGA/AGA, born and discharged appropriate for gestational age; SGA/SGA, born and discharged small for gestational age; AGA/SGA, born appropriate for gestational age and discharged small for gestational age*.

### Comparison Between Groups According to Gestational Age

Characteristics of infants according to the VPI and EPI groups are shown in detail in Table 1. Body composition was measured significantly earlier in infants of the VPI group: 35.3 (SD 1.6) weeks of gestation compared to that of the infants in the EPI group: 37.9 (SD 3.2) weeks of gestation (*p* < 0.001). Weight and length at measurement were significantly higher in the EPI group than those in the VPI group, and there was no significant difference in weight z-score between the groups (Table 1).

The multiple linear regression analysis showed that weight at measurement was not significantly different between the groups (Table 2). FM% was significantly higher in infants in the EPI group than that in infants in the VPI group (Table 2). FFM% was significantly lower in infants in the EPI in comparison to infants in the VPI group (Table 2).

**Table 2:** Body composition values for EPI group and VPI group.

	**Adjusted mean**		**^c^Adjusted mean difference; *p*-value**
	**EPI group**	**VPI group**	**EPI and VPI group**
Weight at scan, g^a^	2,487 (2,394, 2,581)	2,411 (2,346, 2,477)	0.076 (−0.046, 0.199); *p* = 0.217
Fat mass, percentage^b^	17.0 (15.9, 18.1)	15.5 (14.7, 16.2)	1.6 (0.2, 2.9); *p* = 0.034
Fat-free mass, percentage^b^	83.0 (81.9, 84.1)	84.5 (83.8, 85.3)	−1.5 (−2.9, −0.12); *p* = 0.034

a *Mean (95% confidence interval) adjusted for sex and postmenstrual age at scan*.

b *Mean (95% confidence interval) adjusted for sex and postmenstrual age at scan*.

c *p-values for the EPI group in comparison with the VPI group. VPI, very preterm infants ≥28 weeks gestation; EPI, extremely preterm infants <28 weeks gestation*.

### Comparison Between Groups According to Weight Percentile

The characteristics of infants according to the AGA/AGA, SGA/SGA, and AGA/SGA groups are shown in detail in Table 1. Body composition was measured significantly earlier in infants of the AGA/AGA group at 36.2 (SD 2.1) weeks of gestation compared to infants of the AGA/SGA group at 37.9 (SD 2.9) weeks of gestation and infants of the SGA/SGA group at 41.5 (SD 4.5) weeks of gestation (*p* = 0.001). There was no significant difference in length at measurement between the three groups. Weight z-scores at measurement were significantly lower in the SGA groups in comparison to the AGA group, as expected (Table 1).

The multiple linear regression analysis showed that infants in the SGA groups (SGA/SGA and SGA/AGA) had a significantly lower weight at measurement in comparison to the AGA group (Table 3). FM and FFM were not significantly different between the three groups (Table 3).

**Table 3:** Body composition values for the groups according to weight percentile.

	**Adjusted mean**			**^c^Adjusted mean difference; *p*-value**	
	**AGA/AGA**	**AGA/SGA**	**SGA/SGA**	**AGA/AGA vs. AGA/SGA**	**AGA/AGA vs. SGA/SGA**
Weight at scan, g^a^	2,510 (2,458, 2,562)	2,179 (2,067, 2,290)	2,164 (1,917, 2,411)	0.331 (0.205, 0.458) *p* < 0.001	0.346 (0.089, 0.603) *p* = 0.009
Fat mass, percentage^b^	16.2 (15.5, 16.9)	15.4 (13.8, 16.9)	15.5 (12.2, 18.9)	0.8 (−0.9, 2.5) *p* = 0.354	0.6 (−2.9, 4.2) *p* = 0.726
Fat-free mass, percentage^b^	83.8 (83.1, 84.5)	84.6 (83.1, 86.2)	84.5 (81.0, 87.8)	−0.8 (−2.5, 0.9) *p* = 0.354	0.6 (−4.2, 2.9) *p* = 0.726

*AGA/AGA, born and discharged appropriate for gestational age; SGA/SGA, born and discharged small for gestational age; AGA/SGA, born appropriate for gestational age and discharged small for gestational age*.

a *Mean (95% confidence interval) adjusted for sex and postmenstrual age at scan*.

b *Mean (95% confidence interval) adjusted for sex and postmenstrual age at scan*.

c *p-values for the EPI group in comparison with the AGA/AGA group in comparison with the group AGA/SAG and SGA/SGA*.

## Discussion

In this study, we analyzed the body composition in the EPI in comparison to that in the VPI at term-equivalent age. FM percentage was significantly higher in the EPI compared to that in the VPI group. The groups according to weight percentiles (AGA/AGA, AGA/SGA, SGA/SGA, and SGA/AGA) showed no significant differences in FM. Infants born <28 weeks at gestation in comparison to infants born ≥28 weeks of gestation were discharged later. They had a significantly higher percentage of FM% at measurement.

Mean FM% in the whole study cohort (15.9 SD 3.7) corresponds well to published data by Roggero et al. where mean FM% was 14.8 (SD 4.4) in preterm infants <1,500 g at term corrected age (26). Simon et al. conducted a study including 180 preterm infants born <35 weeks of gestation, and the mean FM% was 13.4 (SD 4.2) at term corrected age (27). Furthermore, preterm infants in the study by Kiger et al. had a mean FM% of 17.6 at a median gestational age of 37.1 weeks (28). Body composition reference charts for term and preterm infants according to gestational age at birth and postnatal age at measurement were published very recently (29–33). In comparison to these reference charts, the mean FM% of the present study population was within a normal range (most mean values were >10th and <25th percentile).

The preterm infants in the present study obtained nutrition according to the ESPGHAN 2010 Guidelines (13, 14), using a standard feeding protocol for parenteral and enteral nutrition. The detailed local nutritional strategy was published in 2018 (15). So far, there is no clear evidence-based recommendation for the nutrition of preterm infants according to their body composition (34). Our infants reached an energy intake of 120 kcal/kg/day in the second week after birth compared to 100 kcal/kg/day between the third and fourth week after birth in a previous study (12). Nevertheless, contradictory results in regard to differences in FM% and FFM% in relation to different protein and energy intakes have been described (7, 35). It has been shown that an increase in protein intake and protein to energy ratio might result in an increase in FFM (36, 37). Furthermore, an increase in fat and energy supply resulted in an increase in FM (7).

Roggero et al. published two studies including preterm infants <1,500 g. In these studies, FM% was higher in AGA preterm infants compared to SGA preterm infants (measured at term corrected age) (38, 39). Villela et al. analyzed SGA preterm infants <32 weeks gestational age. They showed that at term corrected age, infants with z-scores <-2 SD had less FM% compared to infants with a z-score >-2 SD (12). In the present study, there was no significant difference in body composition of SGA and AGA preterm infants. The difference may be explained by the fact that we reached higher energy intakes 1–2 weeks earlier. Furthermore, we differentiated between being SGA at birth vs. being SGA at measurement. However, the results of the present study show that there will be the need to further investigate this interesting group of patients in the future.

The strength of the study is the inclusion of extremely preterm infants and the relatively large sample size. Furthermore, we think that the number of patients represent a typical 1 year's cohort for a tertiary neonatal intensive care unit. There are some limitations to the present study, too. First, it is a single center study, and the number of SGA infants is small. Second, single point measurements are difficult to interpret; therefore, longitudinal measurements are of major interest for further studies. Furthermore, the aim of the present study was to investigate the FM% of preterm infants according to gestational age, but at the moment, it is unclear whether this will be the ideal parameter for assessment of neonatal growth. Other parameters might be of equal or even of higher relevance in the future, such as FFM% for instance.

Rochow et al. recently published a longitudinal observational study where the FM% of preterm infants <32 weeks of gestational age [stratified in EPI (<28 weeks) and VPI (28–31 weeks)] had an impact on the neurodevelopment outcome at the age of 18 months. Ninety-six preterm infants received a Bayley III assessment at 18 months corrected age, showing that infants with a higher fat mass during hospitalization had a higher Bayley score (11). In addition, the preterm infants with a lower language score had a notably lower FM% with a significant correlation (11). As there is a positive correlation of FM% and improved neurocognitive outcome in the literature, the nutritional regimen in our cohort seems to be adequate in order to optimize long-term neurodevelopmental outcome in these extremely preterm infants and are of major interest.

The higher FM% in EPI might indicate a higher risk for the development of adiposity and cardiovascular disease in later life, but the optimal body composition of premature infants at term-equivalent age is still unknown; therefore, we can only speculate about that.

In conclusion, EPI had a significantly higher FM% compared to VPI at term-equivalent age. Preterm infants weight percentile at birth and the course to discharge had no impact on body composition. The nutritional regimen in our cohort seems to be adequate as in comparison to reference charts the mean FM% of the present study population was within normal range.

## Data Availability Statement

The raw data supporting the conclusions of this article will be made available by the authors, without undue reservation, to any qualified researcher.

## Ethics Statement

The studies involving human participants were reviewed and approved by Ethics Committee of the Medical University of Graz, Austria. Written informed consent to participate in this study was provided by the participants' legal guardian/next of kin.

## Author Contributions

MB and BU: conception and design. MB, NM, and BW: collection and assembly of data. MB, ZK, CB, NM, and BU: analyses and interpretation of data. MB, CB, and BU: drafting of the article. MB, ZK, CB, NM, BW, SH, and BU: critical revision, editing, and final approval of the article.

### Conflict of Interest

ZK received a scholarship for her Doctoral Studies from the Higher Education Commission (HEC) Pakistan and OeAD. Urlesberger Berndt was a member of the Advisory Board of Milupa/Danone. The remaining authors declare that the research was conducted in the absence of any commercial or financial relationships that could be construed as a potential conflict of interest.

## Abbreviations

AGA: appropriate for gestational age
EPI: extremely preterm infants+
FFM: fat-free mass
FFM%: fat-free mass percentage
FM: fat mass
FM%: fat mass percentage
SGA: small for gestational age
VPI: very preterm infants.
